# Hi-C as a tool for precise detection and characterisation of chromosomal rearrangements and copy number variation in human tumours

**DOI:** 10.1186/s13059-017-1253-8

**Published:** 2017-06-27

**Authors:** Louise Harewood, Kamal Kishore, Matthew D. Eldridge, Steven Wingett, Danita Pearson, Stefan Schoenfelder, V. Peter Collins, Peter Fraser

**Affiliations:** 1 Nuclear Dynamics Programme, The Babraham Institute, Cambridge, CB22 3AT UK; 2 Department of Pathology, Addenbrooke’s Hospital, University of Cambridge, Cambridge, UK; 3 Cancer Research UK Cambridge Institute (CRUK-CI), University of Cambridge, Li Ka Shing Centre, Cambridge, UK

**Keywords:** Hi-C, Chromosome conformation capture, Cancer, Tumour, Glioblastoma, Anaplastic astrocytoma, Chromosome rearrangement, Copy number variation

## Abstract

Chromosomal rearrangements occur constitutionally in the general population and somatically in the majority of cancers. Detection of balanced rearrangements, such as reciprocal translocations and inversions, is troublesome, which is particularly detrimental in oncology where rearrangements play diagnostic and prognostic roles. Here we describe the use of Hi-C as a tool for detection of both balanced and unbalanced chromosomal rearrangements in primary human tumour samples, with the potential to define chromosome breakpoints to bp resolution. In addition, we show copy number profiles can also be obtained from the same data, all at a significantly lower cost than standard sequencing approaches.

**Electronic supplementary material:**

The online version of this article (doi:10.1186/s13059-017-1253-8) contains supplementary material, which is available to authorized users.

## Background

Chromosomal rearrangements are the product of erroneously repaired double strand breaks (DSBs) in DNA resulting in aberrant end joining. Rearrangements can occur via direct exchange, with no gain or loss, of genetic material (reciprocal or balanced rearrangements) or result in deletions or duplications (unbalanced rearrangements). While unbalanced rearrangements can often be detected cytogenetically or with molecular techniques, balanced rearrangements such as inversions and reciprocal translocations, are not detectable using copy number variation (CNV)-based methods and are often cytogenetically cryptic, resulting in a deficit in detection. This means that clinically relevant fusion genes and aberrant juxtapositions of regulatory element with oncogenes are potentially missed. New methods involving next generation sequencing (NGS) have been developed to attempt to overcome this detection bias but none have been unequivocally successful when chromosome breakpoints are not already known [1–12]. One major drawback of using NGS methods to detect balanced rearrangements is the considerable sequencing depth, and associated cost, required to differentiate real breakpoints from false positives caused by sequencing errors. Current methods perform best with at least 40x depth [12], and even then detection can be hampered by low mappability at repetitive regions, meaning that rearrangements involving centromeric, heterochromatic or regions of high homology are often indiscernible. This is a distinct disadvantage as many recurrent rearrangements are mediated by recombination between segmental duplications or homologous sequences [13] and will therefore have at least one breakpoint mapping within repetitive sequences.

Here we demonstrate the power of in-nucleus Hi-C [14], a derivative of the chromosome conformation capture (3C) technique [15], to detect both known and novel, balanced and unbalanced chromosomal rearrangements from cell lines and human tumour samples. In addition to the detection of chromosomal abnormalities, we show that copy number information can also be obtained from the data, allowing gain, amplification and deletion of genomic regions, as well as rearrangements, to be detected from a single experiment. Although Hi-C has previously been used to detect and confirm chromosome rearrangements in cell lines [16–18], it has not, until now, been used on human primary tumour material or to detect copy number information.

## Results

### Balanced and unbalanced translocation detection

In an attempt to detect chromosomal rearrangements and determine accuracy of breakpoint identification we performed in-nucleus Hi-C on two human lymphoblastoid cell lines with known chromosomal translocations between chromosomes 11 and 22. FY1199 has a balanced, constitutional translocation, 46,XY,t(11;22)(q23.3;q11.2), and DD1618 is derived from an Emanuel Syndrome patient (OMIM #609029) carrying an unbalanced product of the same translocation - 47,XX,+der(22)t(11;22)(q23.3;q11.23)mat) [19]. Hi-C interrogates spatial proximity within the nucleus by analysing contacts between genomic regions. Briefly, cells are cross-linked with formaldehyde to preserve spatial juxtapositioning of DNA. The DNA is then cut with a restriction enzyme and free sticky ends are filled in with biotinylated nucleotides prior to religation of fragment ends that are in close spatial proximity. Cross-links are then reversed, the purified genomic DNA fragmented, ligation junctions recovered on streptavidin-coated magnetic beads and the resulting library minimally amplified for paired-end sequencing. For any particular restriction fragment, the vast majority of ligation events will occur with fragments in the first few hundred kilobases (kb) of contiguous sequence in the linear genome. The frequency of such intrachromosomal (*cis*) ligation events, represented by a strong diagonal on Hi-C heatmaps, decreases logarithmically with genomic distance. *Trans*, or interchromosomal, interactions are situated off the diagonal and are typically present at a fraction of the level of *cis* contacts [14].

**Fig. 1:**
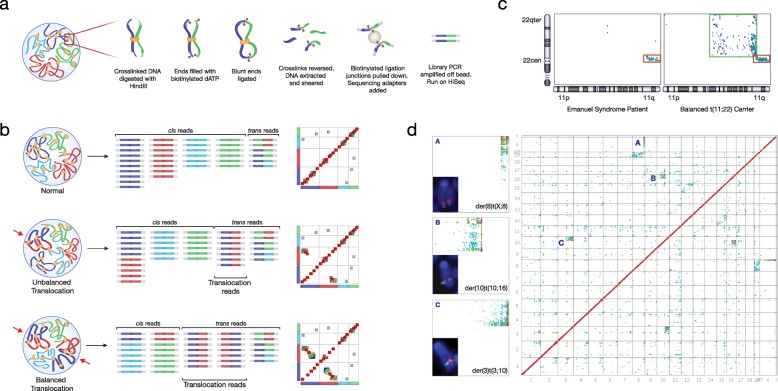
Hi-C detects chromosomal rearrangements. **a** Overview of the Hi-C method. **b** *Cartoon* representation of cross-linked DNA in a normal nucleus (*top*) and both unbalanced and balanced translocation carrying nuclei, with derivative chromosomes (der) demarked. Representative paired end reads and theoretical *heatmaps* are also shown. **c** Partial *heatmaps* for chromosomes 11 and 22 generated from two sets of Hi-C data performed on human cell lines from an Emanuel syndrome patient and balanced translocation carrier. The *red box* outlines interactions observed from the derivative chromosome 22 and the *green box* outlines those from the derivative chromosome 11 (up to the centromere). *Ideograms* for chromosomes 11 and 22 are provided alongside for reference. **d** Hi-C interaction *heatmap* of a mouse cell line showing unsuspected chromosomal rearrangements. Chromosomes are listed along the *x* and *y* axes in numerical order. All three suspected translocations are enlarged and were confirmed by fluorescence *in-situ* hybridisation (FISH), as can be seen by the co-localisation of probes from different chromosomes (one *red* and one *green*) on a single metaphase chromosome (*inset*)

When chromosomal rearrangements bring together distal regions of the same or different chromosomes, distinct blocks of what appear to be unusually strong long-range *cis* or *trans* interactions should be visible on the heatmap (Fig. 1b). Hi-C heatmaps for both cell lines showed clear blocks of strong *trans* ligation between chromosomes 11 and 22. In the unbalanced Emanuel syndrome patient, a single block was present with the strongest contacts occurring at the known breakpoints [20]. In contrast, the balanced translocation cell line, FY1199, showed contacts split between two blocks that produced a ‘butterfly’ appearance (Fig. 1c). These blocks were joined at the point of strongest contacts, corresponding to the known chromosomal breakpoints [20]. This result would be expected when the rearrangement is reciprocal and both derivative chromosomes are present.

### Detection of novel rearrangements

To detect novel rearrangements, we performed in-nucleus Hi-C on a transformed mouse cell line (EKLF^-/-^) [21]. The heatmap showed clear single blocks of strong contacts between sequences on chromosomes 3 and 10, 10 and 16, and X and 8 (Fig. 1d), suggesting unbalanced translocations between these pairs of chromosomes. To confirm these rearrangements, we performed dual-colour DNA fluorescence *in-situ* hybridisation (FISH) on metaphase preparations using probes generated from regions flanking the suspected breakpoints. All three rearrangements were confirmed, proving that Hi-C can detect novel chromosomal rearrangements in cell lines, as also demonstrated by others [16–18].

### Screening of primary human brain tumours

To demonstrate the potential of Hi-C as a method to detect and characterise unknown chromosomal rearrangements in clinical material, we performed Hi-C on six human brain tumours: five glioblastomas (GB) and one anaplastic astrocytoma (AA). These were received as fresh frozen tissue with between 75% and 90% tumour content, as determined by the pathologist. All samples were selected from a larger study and had full ethical approval [22]. Hi-C results revealed dramatic heterogeneity between tumours, from no large scale structural rearrangements detected in one sample (GB183) to rearrangements involving at least 15 of the 24 different chromosomes in another (GB176).

**Fig. 2:**
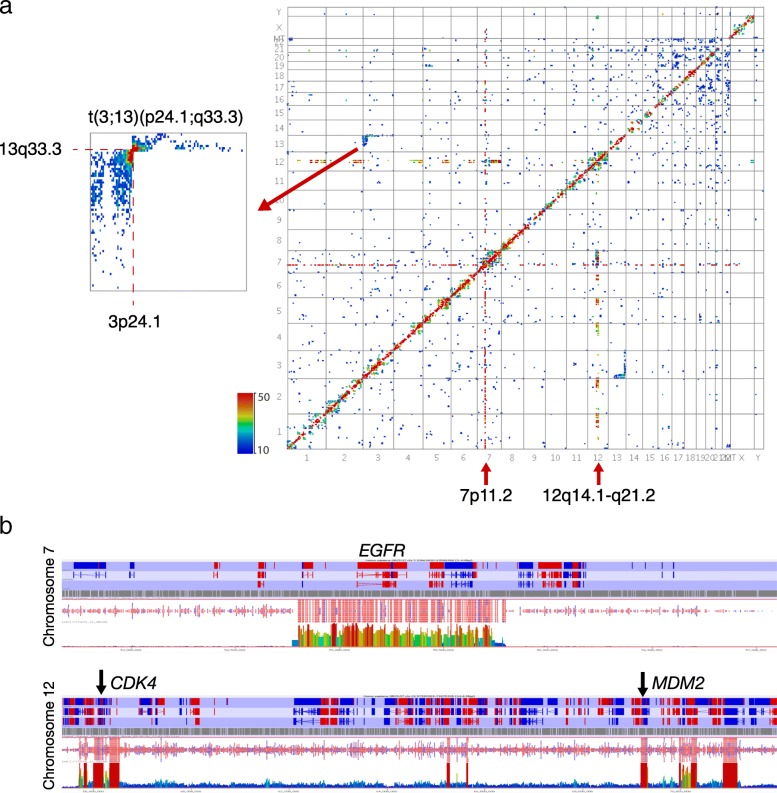
Tumour GB180. **a** *Heatmap* and partial heatmap of tumour GB180 showing a balanced translocation between chromosomes 3 and 13 (t(3;13)(p24.1;q33.3)). *Heatmaps* were coloured by the number of interactions with the colour gradient scaled linearly from ten (*blue*) to 50 (*red*). Bins containing less than ten interactions are not represented. The small *red arrows* indicate amplified regions. **b** Read counts for amplified regions on chromosomes 7 (*top*) and 12 (*bottom*). The high peaks show a significantly higher number of reads than in the surrounding regions. *EGFR*, *CDK4* and *MDM2* oncogenes are labelled

The heatmap from one tumour, GB180, showed the expected strong line of *cis* interactions across the diagonal and also a clear butterfly block of interactions between chromosomes 3 and 13, with the strongest interaction points being in genomic regions corresponding to bands 3p24.1 and 13q33.3, indicating a balanced t(3;13)(p24.1;q33.3) translocation (Fig. 2a). In addition to this chromosomal rearrangement, there was also a distinct line of interactions from a small region of chromosome 7 to regions throughout the genome. This was suggestive of amplification via double minutes – small extrachromosomal DNA fragments that commonly contain oncogenes and are spread throughout the nucleus [23]. Sequencing reads from chromosome 7 revealed a highly amplified 1 Mb region corresponding to the line on the heatmap, with the read count for this region being substantially higher than the rest of the chromosome. This region contained the *EGFR* oncogene, known to be amplified in glioblastoma, with around 42% of cases showing amplification of this gene via double minutes [24]. *EGFR* amplification was also seen in tumours GB176 and GB182. In addition to the chromosome 7 amplification, the heatmap for tumour GB180 also showed a similar pair of lines situated close together on chromosome 12. These represented additional oncogene containing regions that are amplified in glioblastoma, with *CDK4* being in one and *MDM2* (murine double minute homolog 2) in the other [25, 26] (Fig. 2b).

**Fig. 3:**
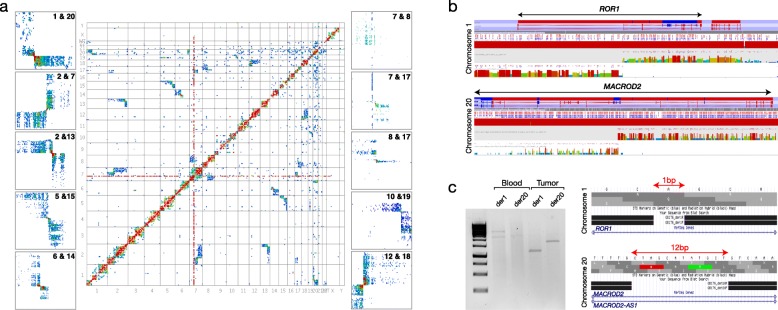
Tumour GB176. **a** *Heatmap* and partial heatmaps of tumour GB176 showing some of the rearrangements present in this tumour. **b** Hi-C ‘other ends’ from regions distal and proximal to the suspected breakpoint on chromosome 1 (*top*) and chromosome 20 (*bottom*) showing the breakpoint regions. A sudden drop-off in the number of reads can be seen where the remaining chromosome is not involved in the translocation and is therefore not in *cis*. **c** *Left*: Polymerase chain reaction (PCR) on tumour and blood DNA from GB176 showing amplification products from both derivative chromosomes, indicating a balanced translocation. *Right*: BLAT results from sequenced tumour specific PCR amplicons showing the breakpoint regions on chromosome 1 (*top*) and 20 (*bottom*). The gaps in the BLAT results show deletions at the translocation breakpoints

While GB180 showed only one translocation, glioblastoma GB176 was more complex and showed evidence of multiple chromosomal rearrangements, the majority of which showed the butterfly pattern associated with balanced translocations (Fig. 3a). For example a t(1;20)(p13.1;p12.1) translocation could be seen, as could a t(5;15)(q32;q22.31), t(2;13)(q34;q31.1) and t(10;19)(q25.1;q13.33). Balanced translocations could also be seen in other tumours, such as a t(9;11)(q32;q13.2) in GB238 and a t(X;16)(p11.22;q22.1) in AA86 (Additional file 1: Figures S1–S4). In addition, derivative chromosomes generated from unbalanced translocations could be seen in the anaplastic astrocytoma sample, AA86. These present as single blocks of interactions, in this case chromosomes 9;11 and 10;18, as opposed to the butterfly appearance of balanced rearrangements (Additional file 1: Figure S4).

Some tumour heatmaps showed chromosomes that were involved in rearrangements with more than one partner chromosome. As there are generally more than one of each chromosome per cell, it may be that each is involved in separate rearrangements—for example the 2;7 and 2;13 rearrangements in GB176 do not seem to be associated as they share no common interaction blocks or breakpoints. However, in cases where breakpoints appear to be the same, or where interaction blocks appear between multiple chromosomes (e.g. regions of chromosomes 7, 8 and 17 all interact with each other in GB176; see Additional file 1: Figure S5), it is likely that complex, three-way rearrangements are occurring. This situation could also be seen in tumours GB182, GB238 and AA86 (Additional file 1: Figures S1, S3 and S4).

Some rearrangements, such as the 6;14 and the 12;18 in GB176, appeared to be complex and involve inversions at the breakpoints. In these cases, the highest number of interactions were offset from the connecting point of the ‘butterfly’. In addition to apparent inversions, there was evidence, in the form of gaps in interaction blocks or a sudden drop-off in interactions, of deletions having also occurred. For example, the 6;14 rearrangement showed a sudden drop-off in interactions on chromosome 6q and gaps in both interaction blocks, suggesting deletions on both derivative chromosomes (Additional file 1: Figure S6). Similar gaps could also be seen in the 7;17 and 8;17 rearrangements in GB176, giving the interaction blocks a striking striped appearance.

One rearrangement in GB176, namely, the t(1;20)(p13.1;p12.1), was examined in more detail. By selecting the connecting points of the butterfly on the heatmap, approximate breakpoint coordinates were surmised. Analysing interactions from regions just proximal/distal to these showed expected *cis* interactions but also *trans* interactions on the partner chromosome of the rearrangement. At a certain point, the *trans* interactions dropped off suddenly due to the remainder of that chromosome not being involved in the translocation (Fig. 3b). This allowed breakpoints to be determined to within one or two HindIII fragments. In the t(1;20), the chromosome 1 breakpoint was within a single restriction fragment, approximately 1.2 kb in size (chr1:64471372-64472588, GRCh37), within the *ROR1* gene. The chromosome 20 breakpoint was within two adjacent restriction fragments (chr20:14895015-14895976 and chr20:14895977-14903670, GRCh37), a region of approximately 8.6 kb in size within an intron of the large *MACROD2* gene.

To attempt to map the breakpoints to bp resolution, we designed polymerase chain reaction (PCR) primers to amplify the suspected breakpoint regions on chromosomes 1 and 20. By combining forward and reverse primers from different chromosomes, a product could only be obtained if the relevant derivative chromosomes were present. Also, to confirm that the rearrangement was tumour-specific and not constitutional, DNA from the tumour was run alongside that from peripheral blood of the same patient. Amplification of the normal chromosomes could be seen in both sets of DNA but tumour DNA also generated products for both derivative chromosomes 1 and 20. Sequencing of the PCR fragments identified breakpoints within intron 1 of *ROR1* and intron 4 of *MACROD2* (also falling within *MACROD2-AS1*, an antisense RNA of the gene) and showed that, compared with the reference sequence, a deletion of 1 bp had occurred at the breakpoint on chromosome 1 (chr1:64472097, GRCh37) and 12 bp had been deleted on chromosome 20 (chr20:14895406-14895417, GRCh37) (Fig. 3c). The result of this balanced translocation is therefore a reciprocal fusion between the *ROR1* and *MACROD2* genes.

#### Generation of linkage score plots

**Fig. 4:**
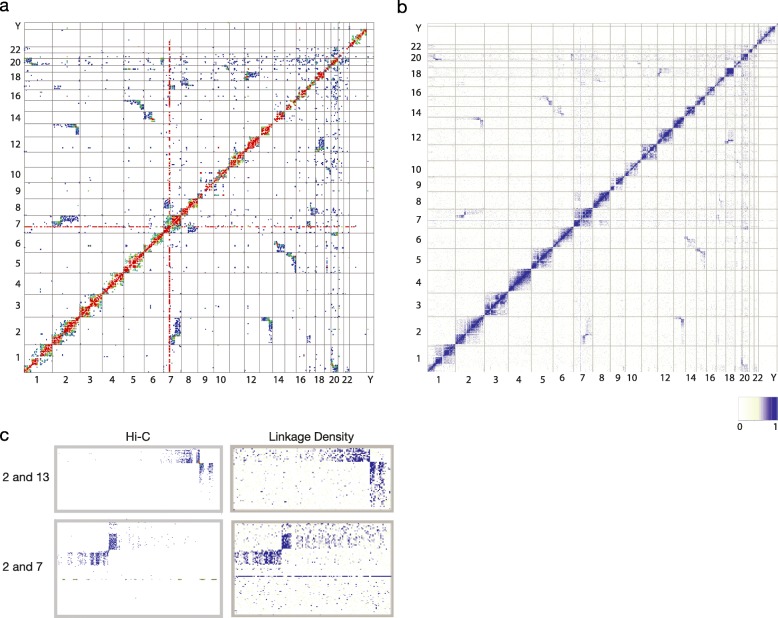
Hi-C and normalised linkage density heatmaps for tumour GB176. **a** Hi-C interaction *heatmap* generated using 500 kb probe size. **b** *Heatmap* of normalised linkage densities at 500 kb resolution. **c** Examples of enlarged regions of both heatmaps showing the rearrangements involving chromosomes 2 and 7 (*left*) and chromosomes 2 and 13 (*right*)

To determine whether we could confirm the presence of rearrangements using an approach other than visual inspection of the number of interactions on a Hi-C heatmap, we generated linkage density plots for the Hi-C data in a method similar to the one Burton et al. used to validate translocations in the HeLa cell line [16]. To do this, we split the genome into bins of approximately 500 kb and computed pairwise interaction scores among all bins. To correct for Hi-C biases that occur due to reads only being available within a certain distance of HindIII restriction sites, each interaction score was normalised by the number of HindIII sites contained within that bin. This produced a linkage score for each bin to every other bin within the genome and allowed those bins with high linkage scores to be determined. These high scoring bins were those situated closely in *cis* (as would be expected) and also blocks of bins that had higher scores than surrounding areas. These matched with suspected rearrangements from the Hi-C interaction heatmaps and bins with the highest scores were situated at/near to the suspected rearrangement breakpoints. All of these bins represent linkage density scores greater than the 99th percentile of overall linkage densities (the top 1% of values). For ease of comparison, normalised linkage densities were plotted into genome-wide chromosome heatmaps, similar to those obtained from standard Hi-C interaction data (Fig. 4 and Additional file 1: Figures S7 and S8). In this initial study, rearrangements were determined by visual inspection of interaction heatmaps and linkage plots, where rearrangements between chromosomes could clearly be determined. In the linkage data, these rearrangements could also be seen as multiple consecutive interchromosomal bins of linkage scores in the top 1% of all values. Work is now underway to develop an algorithm to computationally detect these rearrangements.

Tumour GB176 showed a number of regions with high normalised linkage densities, many of which appeared on the heatmap to have a similar ‘butterfly’ appearance to those seen on the Hi-C interaction heatmap. Lines showing high linkage scores could also be seen on chromosome 7. Placing the linkage plot next to the Hi-C interaction heatmap showed that rearrangements suspected from visual inspection of Hi-C interaction heatmaps could be confirmed computationally, via calculation of normalised linkage matrices across the genome (Fig. 4). Similar confirmatory results were seen for the other five tumours (Additional file 1: Figures S7 and S8).

#### Copy number information

In addition to chromosome rearrangements, copy number changes are both prevalent and important in cancer. To determine whether we could extract copy number information from the Hi-C data we had already generated, we treated it in a manner similar to shallow whole-genome sequencing (sWGS), though with some modifications to the Hi-C data, and processed both sets of data through the same copy number pipeline (QDNAseq). QDNAseq provides copy number information from DNA samples without the requirement for a reference and includes steps to correct for issues caused by mappability and GC content and also blacklists a set of genomic regions known to be problematic in copy number analyses. The output of QDNAseq is read counts per bin, which have been corrected, filtered, normalised and log_2_-transformed [27].

As mentioned above, due to the nature of Hi-C data, only regions of the genome that are situated around HindIII restriction sites will be captured. This introduces a bias into sequencing data obtained via Hi-C as compared to standard sWGS data. To correct for this, once the Hi-C data had been run through the QDNAseq pipeline, each resulting bin was divided by the number of HindIII restriction sites that it contained, effectively normalising for this bias.

For the six tumour samples, segmented QDNAseq outputs (autosomes only) obtained from Hi-C and sWGS data were compared to determine their concordance. At a bin size of 100 kb, the two sets of data showed correlation coefficient (r) values in the range of 0.93–0.99 (*p* < 0.01) (Table 1), with r values between non-related samples not exceeding 0.68 (Additional file 1: Figure S9). In order to exclude any regions that showed consistent large changes between the two sets of results, the difference between Hi-C and sWGS output values was determined for each bin and the total difference (i.e. the sum of the differences for all six tumours) calculated. Two different thresholds of exclusion were applied to the data – namely the 99.9th and 99.5th percentiles – with all values above these being excluded from correlation analyses. The 99.9th percentile cutoff removed 31 of 28,822 100 kb bins (Additional file 2: Table S1) and produced r values in the range of 0.94–0.99 (*p* < 0.01) for segmented outputs (Table 1 and Additional file 1: Figure S10). There were 155 bins above the 99.5th percentile cutoff (Additional file 3: Table S2) and r values for segmented outputs did not differ from above (Table 1 and Additional file 1: Figure S11). These excluded regions do not therefore significantly contribute to noise in the Hi-C samples and only marginally affect the correlation between the Hi-C and sWGS QDNAseq data. We therefore decided to remove only the most variable regions and used the 99.9th percentile for our data (Additional file 4: Table S3).

**Table 1:** Correlation coefficients for Hi-C versus sWGS QDNAseq data with and without filtering

Tumour	r values	r values - 99.9th percentile cutoff	r values - 99.5th percentile cutoff
AA86	0.98	0.98	0.98
GB182	0.97	0.97	0.97
GB176	0.99	0.99	0.99
GB238	0.97	0.98	0.98
GB180	0.98	0.98	0.98
GB183	0.93	0.94	0.94

Using the 99.9th percentile cutoff, QDNAseq results using Hi-C data and those using sWGS were highly concordant. Five of the six samples had r values of 0.97 or higher with one sample being slightly lower (r = 0.94 in GB183). Glioblastomas are highly heterogeneous cancers with considerable genetic heterogeneity observed between multiple sampling sites from within the same tumour [28]. It should be noted that while the samples taken for Hi-C and sWGS, were obtained from the same piece of excised tumour, they were collected from different sampling sites leaving open the possibility that tumour heterogeneity could explain the slightly lower correlation values in tumour GB183.

We show that Hi-C data can be used to detect alterations in copy number, without the need for a reference, using the QDNAseq pipeline, with only slight modifications to correct for inherent Hi-C biases. Copy number analyses of the six brain tumours using both sWGS and Hi-C confirmed amplifications of the *EGFR* region on chromosome 7 in GB176, GB180 and GB182, as suggested by the Hi-C interaction data. The amplifications of chromosome 12 in GB180 were also confirmed. Gain of chromosome 7, a hallmark of glioblastomas [25, 26, 29], was detected in all glioblastoma samples (those with a GB prefix) but not the anaplastic astrocytoma, AA86. Other known aberrations, such as loss of chromosome 10, were also observed and deletion of the tumour suppressor gene *CDKN2A* on chromosome 9p21.3, was seen in all tumours except GB180 (Additional file 4: Table S3).

## Discussion

This is the first report of Hi-C as a tool to detect both chromosomal aberrations and copy number in primary human tumour material. While Hi-C has previously been used to detect and confirm rearrangements in cell lines [16–18], it has not, until now, been used in a way that has potential therapeutic and clinical implications. Hi-C on these six primary tumour samples revealed amplifications of known oncogenes, deletions of a tumour suppressor gene and many structural rearrangements, both balanced and unbalanced. One balanced rearrangement studied in detail was shown to result in the fusion of two genes known to be involved in cancer (*MACROD2* [30–32] and *ROR1* [33–35]).

We show that from a single Hi-C assay, information on both chromosome rearrangements and copy number changes can be obtained, without the requirement for deep sequencing (see Additional file 5: Table S4). The large blocks of interactions seen in Hi-C heatmaps also provide an overall picture as to what is happening with whole chromosomes as opposed to just information about any breakpoint regions. The ability to determine structural and copy number aberrations along with the ‘bigger picture’ that Hi-C provides could prove a powerful aid in the identification and understanding of the complex chromosomal rearrangements often seen in cancer.

Unlike standard cytogenetic G-band preparations, Hi-C does not rely on the presence of dividing cells and can be used on all nucleated cell types. It is therefore a powerful tool in the analysis of solid tumours, where cytogenetic analysis is difficult and rarely performed as part of routine diagnosis/analysis, yet fusion genes can play a critical clinical role [36, 37]. Hi-C allows these tumours to be interrogated and provides a means to alleviate the bias in detection of both chromosomal rearrangements and fusion genes towards blood borne cancers.

Although NGS sequencing is now widely being used to screen for chromosomal rearrangements, a high degree of sequence depth is required to enable the exclusion of false positives, with efficacy of detection decreasing with decreasing coverage [12, 38]. In contrast to standard sequencing approaches used to detect balanced chromosomal rearrangements, Hi-C does not rely on the presence of breakpoint spanning reads. The strength that Hi-C has over other techniques is that it uncovers large blocks of multiple interactions occurring between one chromosome and another. This is due to the regions either side of the breakpoint being situated in *cis* and having a much higher interaction frequency than would be expected if they were truly in *trans*. These large blocks of interactions also provide an overall picture as to what is happening along the length of the chromosomes involved in the rearrangements, as opposed to just information from a small region around the breakpoints. This enables more complex rearrangements to be observed. The presence of multiple interactions, instead of a small number of breakpoint spanning reads, means that even with a relatively low number of reads and resulting low sequence coverage (the highest coverage in the samples outlined here was 0.55x; see Additional file 5: Table S4), rearrangements can still be observed. Hi-C allows the problematic detection of rearrangements that involve poorly mappable or repetitive regions to be overcome, as surrounding regions that can be mapped will still show the chromosomal interactions indicative of a rearrangement.

Due to high sequence coverage not being required, Hi-C costs significantly less than deep WGS. Although Hi-C library prep costs are higher, overall Hi-C costs are still less than one-third of those for deep (approximately 30x) WGS (Table 2), which results in a saving of over £900 per sample in our hands.

**Table 2:** Comparison of sWGS, Hi-C and deep WGS costs

	Costs (£)^a^			Rearrangement detection?	Copy number detection?
	Library prep	Sequencing	Total per sample		
sWGS	71	30–82	101–153	N	Y
Hi-C	165	211	376	Y	Y
Deep WGS	44	1270	1314	Y	Y

^a^Based on in-house protocols and costs using the Illumina HiSeq 4000. sWGS prepared using Illumina Nextera DNA library kit and 20–50 million 50 bp single end reads. Hi-C sequencing costs based on six samples per lane, 150 bp paired end. Deep WGS prepared using Illumina TruSeq DNA PCR-Free and one lane of 150 bp paired end sequencing (approx. 30x coverage)

## Conclusions

In summary:

- Hi-C can be used to detect both balanced and unbalanced chromosome rearrangements
- The same Hi-C data can be used detect copy number changes
- Detection of rearrangements using Hi-C does not require deep sequencing
- Rearrangements involving poorly mappable regions can be detected
- Hi-C provides information about whole chromosomes involved in rearrangements, not just the breakpoints themselves
- Hi-C does not require dividing cells and can be used on all nucleated cell types
- Hi-C costs significantly less than deep WGS

Hi-C has the ability to play a pivotal role in the detection of novel chromosomal abnormalities, both balanced and unbalanced, and the potential discovery of new fusion genes. The technique requires extremely low coverage compared with other NGS techniques being used for this purpose and has the additional advantage of being able to provide copy number information from the same data. Further use of Hi-C in this way and the generation of additional bioinformatic pipelines to analyse the data should cement the use of the technique for the detection of chromosomal rearrangements from all nucleated cell/tissue types and establish its role in clinical research.

## Methods

### Cell culture

The transformed mouse cell line [21] was grown in Dulbecco’s modified eagle medium (DMEM) with 10% fetal calf serum (FCS) and 1% penicillin/streptomycin supplemented with puromycin. Human lymphoblastoid cell lines (FY1199 and DD1618 - European Collection of Cell Cultures (ECACC)) were grown in RPMI-1640 medium with 10% FCS and 1% penicillin/streptomycin.

### Hi-C on cell lines

Hi-C on cell lines was performed in nucleus as outlined in Nagano et al*.* [14]. Fifty basepair paired end sequencing was performed on the Illumina HiSeq 1000 instrument for human samples and 125 bp paired end sequencing on the Illumina HiSeq 2500 was performed for the mouse cell line. Hi-C data were filtered using the HiCUP pipeline v0.5.8 [39] (http://www.bioinformatics.babraham.ac.uk/projects/hicup/) and output data imported into the Babraham Bioinformatics SeqMonk program (http://www.bioinformatics.babraham.ac.uk/projects/seqmonk/). Interaction heatmaps were generated using either 1 Mb or 500 kb probes and Hi-C read count quantitated using default settings. Once the heatmap was generated, the Min Absolute count was increased to ten to reduce background noise on the heatmaps and to enrich for interaction blocks over single interactions. This value could be further increased if required. Heatmaps were coloured by the number of interactions with the colour gradient scaled linearly from blue to red, with blue representing the minimum absolute count specified (see above—ten as default for heatmaps in this manuscript) and red denoting a fixed value of 50. Bins containing no interactions or a number of interactions less than the minimum specified are not represented on the heatmaps.

### Hi-C on tumour samples

Tumours were received from Prof. V. Peter Collins (Department of Pathology, Addenbrooke’s Hospital, Cambridge, UK), with accompanying full ethical approval, as fresh frozen pieces consisting of between 75% and 90% tumour content, as determined by the pathologist. Approximately 160 mg of frozen tumour was finely chopped before being fixed and taken through the Hi-C protocol as described above.

### FISH on cell lines

Fixed cell suspensions were prepared for FISH. Colcemid (Gibco® KaryoMAX®) was added to the culture medium to a final concentration of 0.1 μg/mL (1 in 100) and the flask incubated at 37 °C for 1 h. Cells were spun and supernatant discarded. Ten millilitres prewarmed hypotonic solution was added (for human: 1:1 1% (w/v) sodium citrate: 0.56% (w/v) (0.075 M) KCl and for mouse: 0.56% (w/v) (0.075 M) KCl only) and incubated at 37 °C for 12 min. Cells were pelleted, the supernatant discarded and the cells washed with and then stored (at –20 °C) in fresh 3:1 methanol: acetic acid fix. Bacterial artificial chromosomes (BACs) were obtained from BACPAC Resource Center (BPRC) at the Children’s Hospital Oakland Research Institute. Clones were grown and DNA extracted according to BPRC protocols. BAC DNA was labelled using ARES™ Alexa Fluor® Labelling Kits (Alexa Fluor® 488 and Alexa Fluor® 594) according to the manufacturer’s protocol. FISH was performed on fixed cell suspensions according to standard methods [40, 41].

### Breakpoint sequencing

Normal PCR was performed using standard conditions. PCR products were purified using the Qiagen QIAquick PCR purification kit, according to the manufacturer’s instructions, and the resulting products Sanger sequenced.

### QDNAseq – sWGS

DNA was extracted from tumour tissue using the Qiagen QIAamp DNA Micro Kit, according to manufacturer’s instructions. Sequencing libraries were then prepared according to Scheinin et al. [27] and resulting libraries sequenced (50 bp single end) on an Illumina HiSeq 2500. The data were then run through the QDNAseq Bioconductor package (v.1.8.0) using default variables and a 100 kb bin size. Sex chromosomes were not analysed.

### QDNAseq – Hi-C

Hi-C paired end raw sequencing reads were truncated through HiCUP v0.5.8. The truncated FASTQ files were mapped to the human reference genome (GRCh37) using bowtie2 (v2.2.8). The forward reads bam files were merged with reverse reads bam files (using only mapped reads from reverse reads bam file). The merged bam files were run through the QDNAseq Bioconductor package (v.1.8.0) and copy number identified by binning the reads in 100 kb windows. Thereafter, Hi-C corrections were applied on each bin for the number of HindIII restriction sites.

### Linkage plots

Hi-C paired end raw sequencing reads were processed through HiCUP v0.5.8 by mapping to the human reference genome (GRCh37) using bowtie2 (v2.2.8). The pairwise interaction matrix for each sample was computed using 500 kb windows. Each bin of interaction matrix was normalised by the number of HindIII restriction sites in each bin and plotted to generate linkage density plots.

## Additional files

Additional file 1: Supplementary **Figures S1–S11.** (PDF 14694 kb)

Additional file 2: Table S1. QDNAseq bins having total difference values falling above the 99.9th percentile. (XLSX 15 kb)

Additional file 3: Table S2. QDNAseq bins having total difference values falling above the 99.5th percentile. (XLS 101 kb)

Additional file 4: Table S3. QDNAseq outputs for sWGS and corrected Hi-C inputs for the six tumours. (XLS 14773 kb)

Additional file 5: Table S4. Read counts and sequence coverage of processed samples and **Table S5**: GEO sample accession numbers. (DOCX 90 kb)

## Abbreviations

3C: Chromosome conformation capture
AA: Anaplastic astrocytoma
bp: Basepair
CGH: Comparative genomic hybridisation
CNV: Copy number variation
DSB: Double strand breaks
FISH: Fluorescence *in-situ* hybridisation
GB: Glioblastomas
kb: Kilobases
Mb: Megabase
mg: Milligrams
NGS: Next generation sequencing
sWGS: Shallow whole-genome sequencing
WGS: Whole-genome sequencing
