# Supplementary material for: Hi-C as a tool for precise detection and characterisation of chromosomal rearrangements and copy number variation in human tumours
Source: Genome Biol. 2017 Jun 27;18:125. doi: 10.1186/s13059-017-1253-8 (PMC5488307; doi:10.1186/s13059-017-1253-8)
Supplement: Supplementary file 1 — Supplementary Figures S1–S11. (PDF 14694 kb) [file 13059_2017_1253_MOESM1_ESM.pdf]

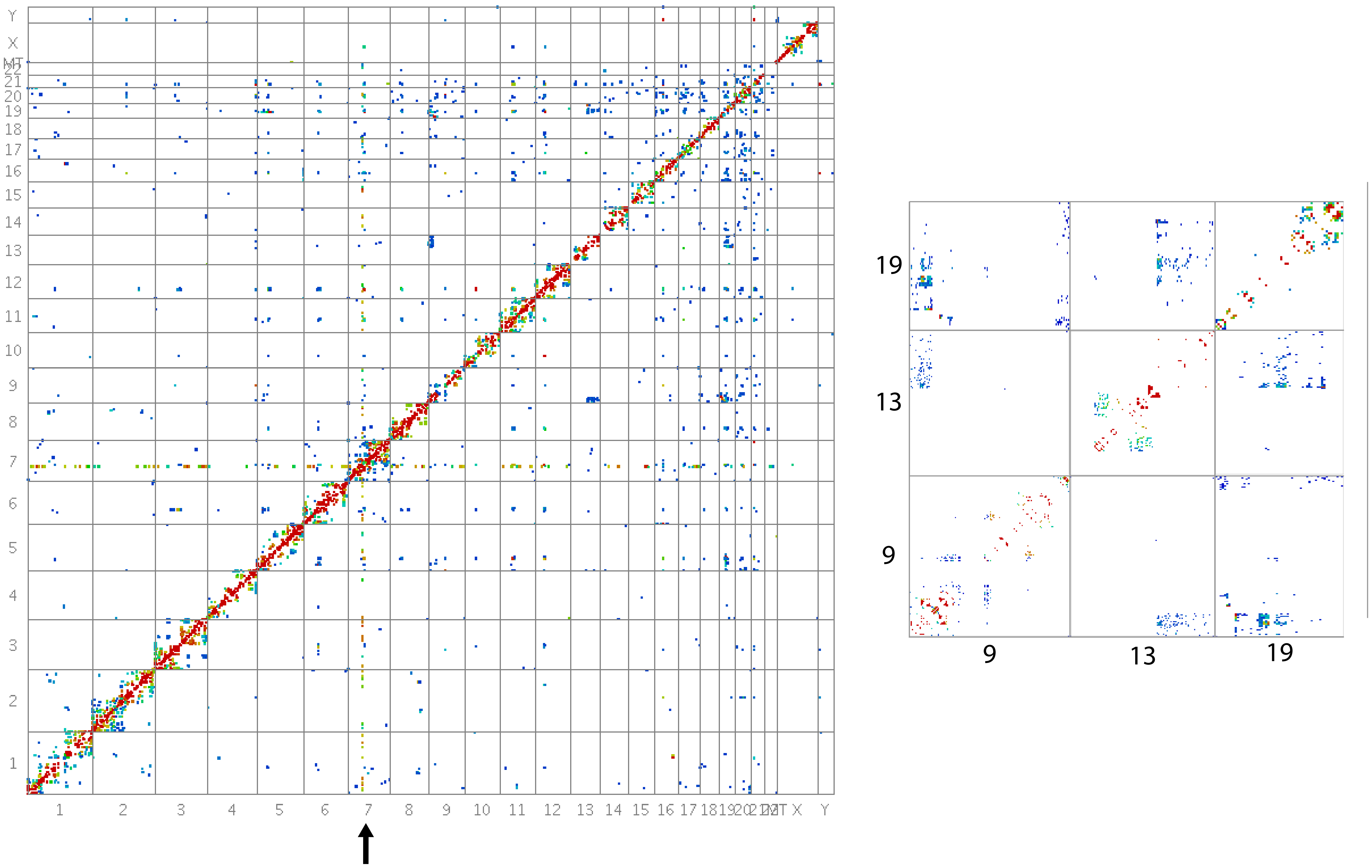

Figure S1: Tumour GB182.
Hi-C interaction heatmap for tumour GB182 showing interactions between chromosomes 9, 13 and 19 (enlarged in the partial heatmap on the right) and amplification of the EGFR gene region (black arrow).

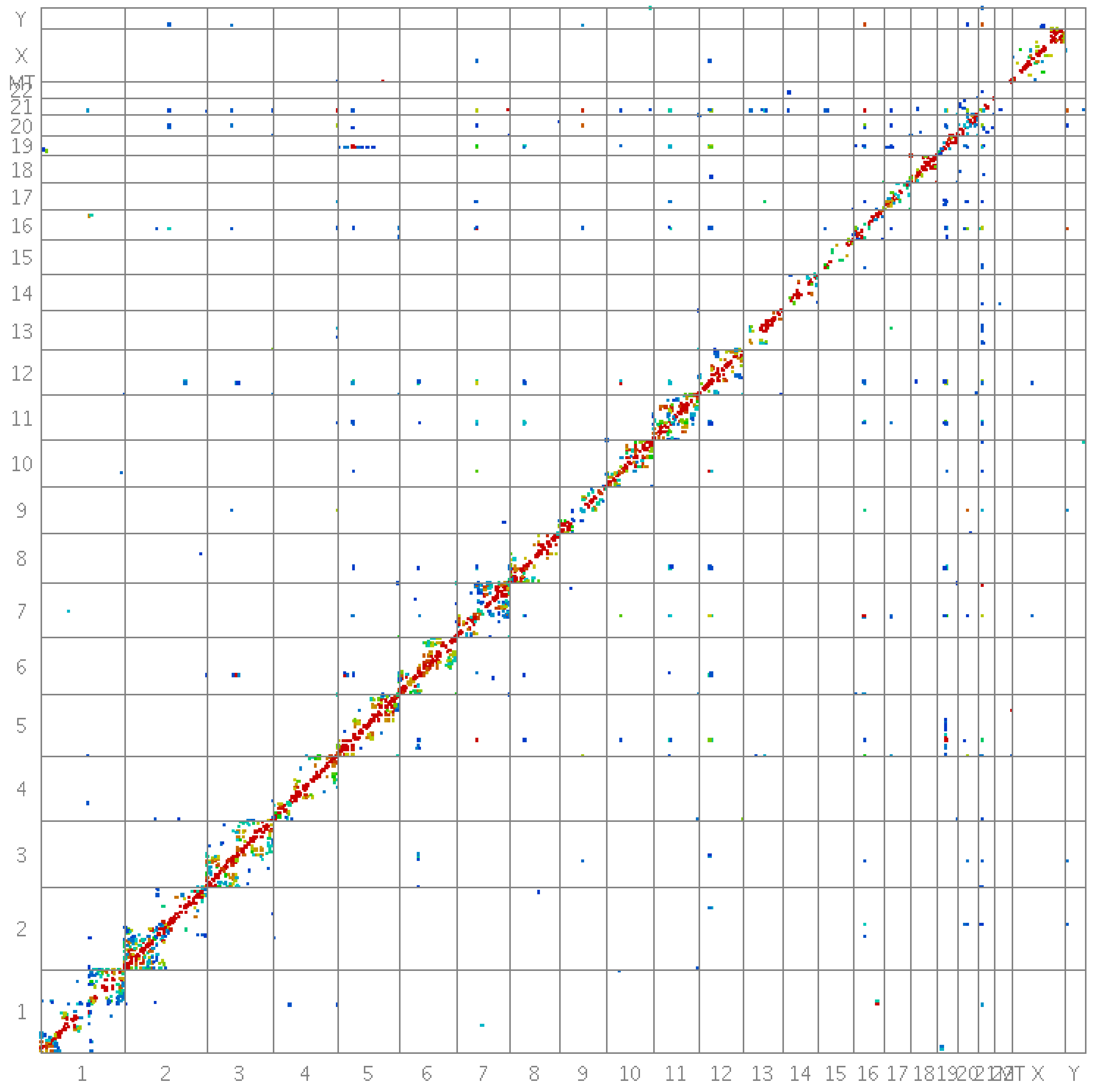

Figure S2: Tumour GB183.
Hi-C interaction heatmap for tumour GB183

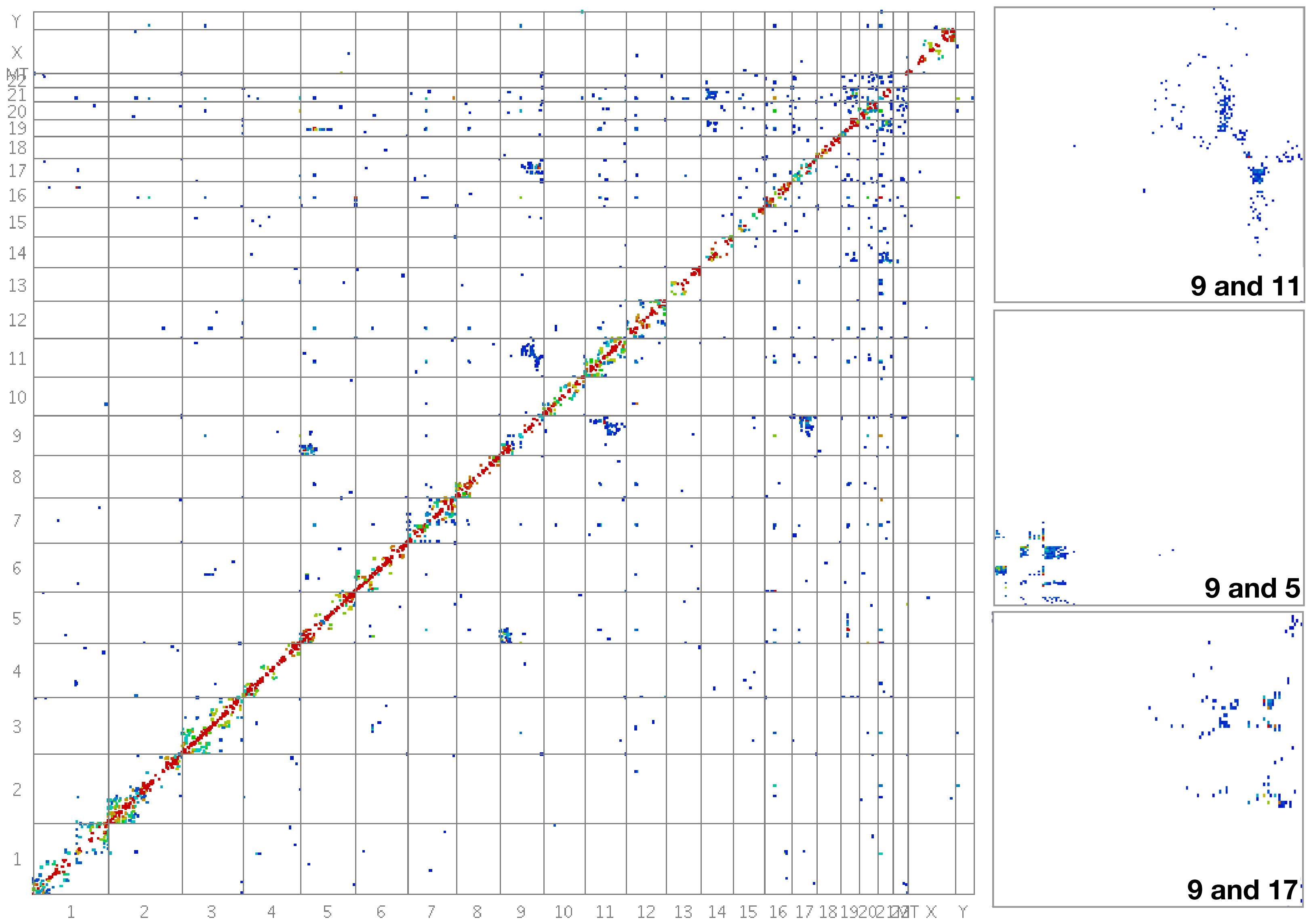

Figure S3: Tumour GB238.
Hi-C interaction heatmap and partial heatmap for tumour GB238. The enlarged boxes on the right show interactions between chromosomes 9 and 11 suggestive of a balanced translocation t(9;11)(q32;q13.2) and interactions between chromosome 9 and chromosomes 5 and 17.

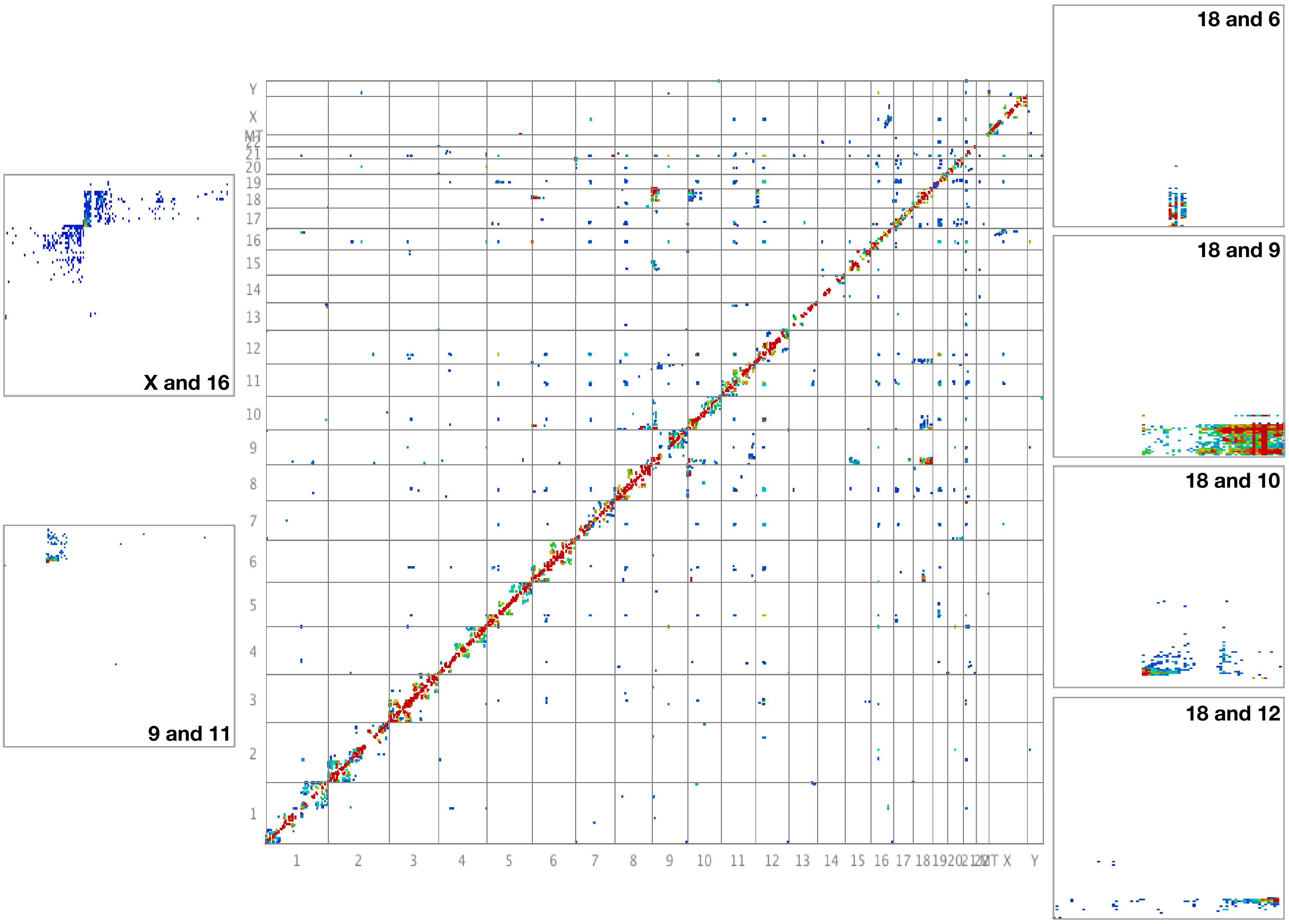

Figure S4: Tumour AA86.
Hi-C interaction heatmap and partial heatmap for tumour AA86. The enlarged boxes on the left show interactions between chromosomes X and 16 suggestive of a balanced translocation t(X;16)(p11.22;q22.1) (top) and derivative chromosomes formed from unbalanced translocations namely der(9)t(9:11)(p21.2;q23.2) (bottom). Boxes on the right show interactions between chromosome 18 and four different chromosomes.

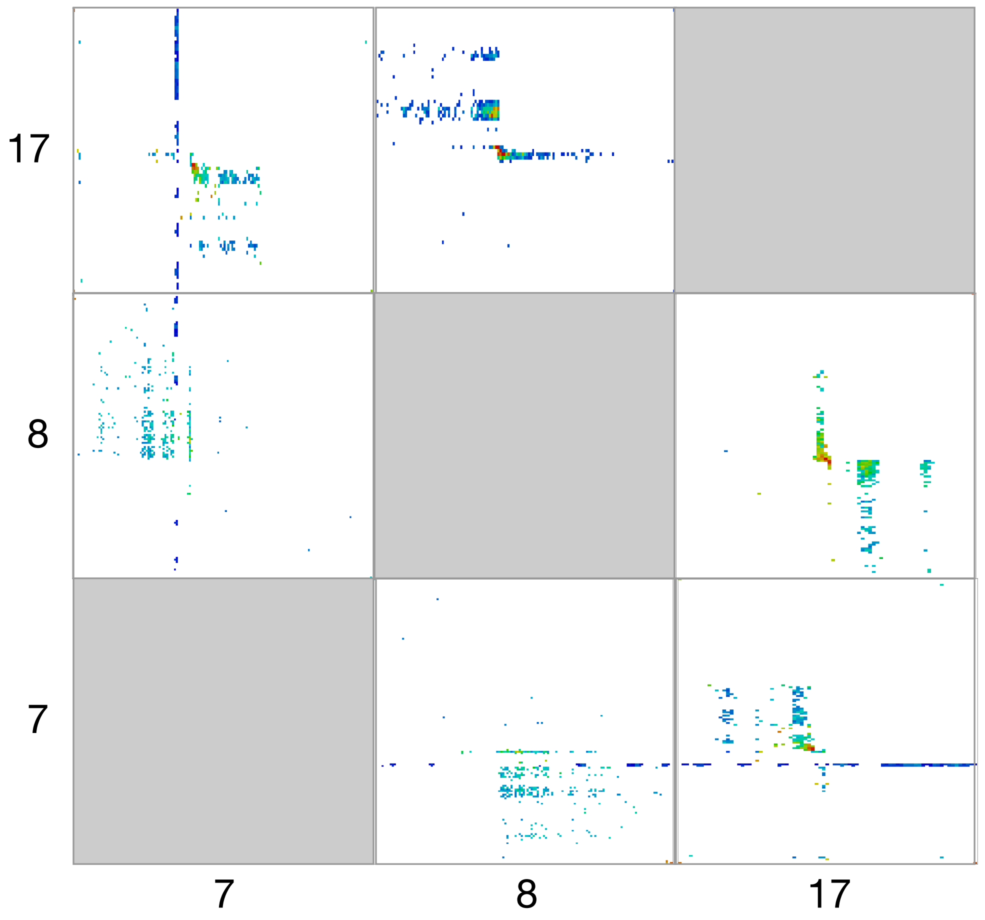

Figure S5: Partial Hi-C interaction heatmaps for chromosomes 7, 8 and 17 for tumour GB176.
Partial Hi-C heatmaps showing interactions between chromosomes 7, 8 and 17 in tumour GB176. The presence of interactions for the same regions between all chromosomes suggests a 3-way rearrangement.

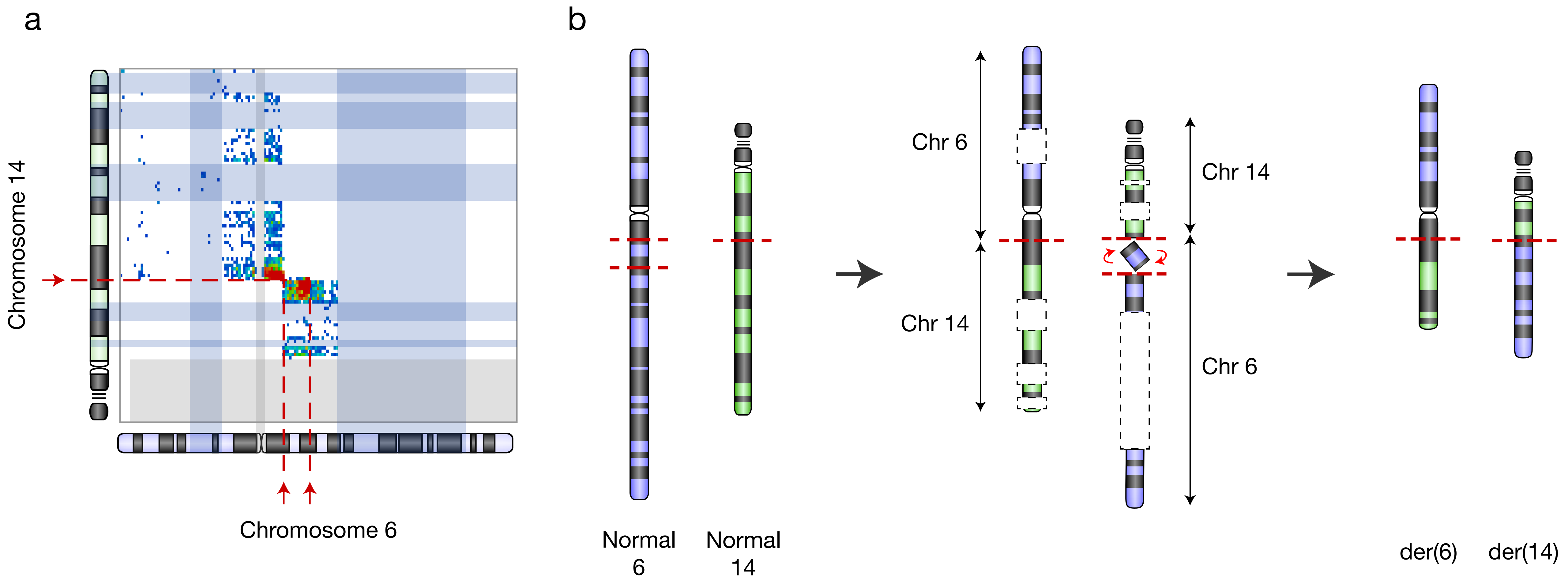

Figure S6: Partial Hi-C interaction heatmap and chromosome ideograms for chromosomes 6 and 14 in tumour GB176.
a) Partial Hi-C heatmaps showing interactions between chromosomes 6 and 14 in tumour GB176. Shaded blue areas show regions that appear, due to the drop off in interactions, to be deleted on the translocated chromosomes. Grey shaded regions represent the centromeres. Red dotted lines show the suspected breakpoint regions for the translocation and the inversion region on chromosome 6. b) Ideograms for chromosomes 6 and 14 showing breakpoints (red lines), inversion (red arrows), suspected deleted regions (dashed white boxes) and the resulting derivative (der) chromosomes.

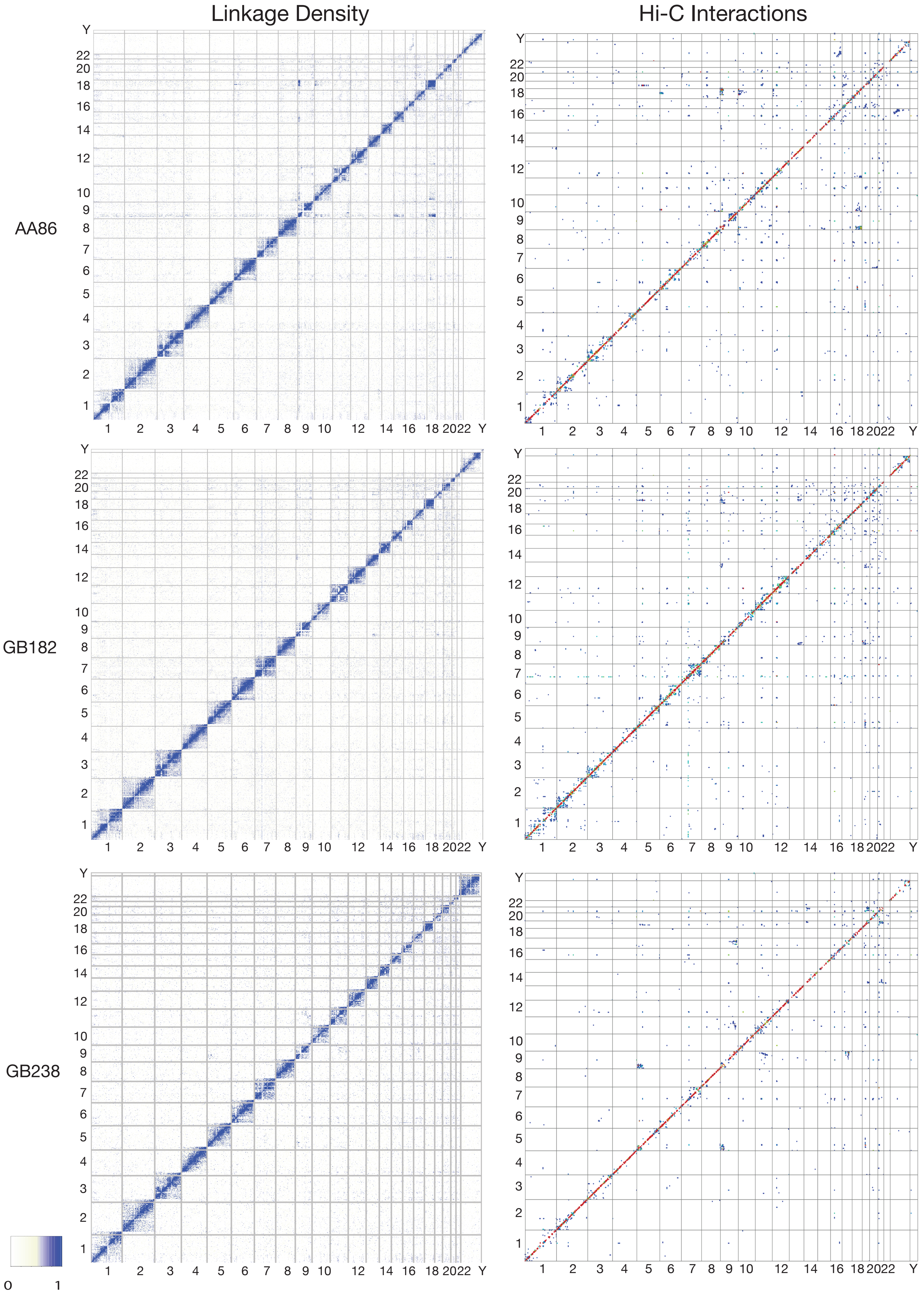

Figure S7: Linkage density plots and Hi-C interaction heatmaps for tumours AA86, GB182 and GB238. Plots of normalised linkage densities (left) and Hi-C interaction heatmaps generated using 500 kb probes (right) for tumours AA86 (top), GB182 (middle) and GB238 (bottom).

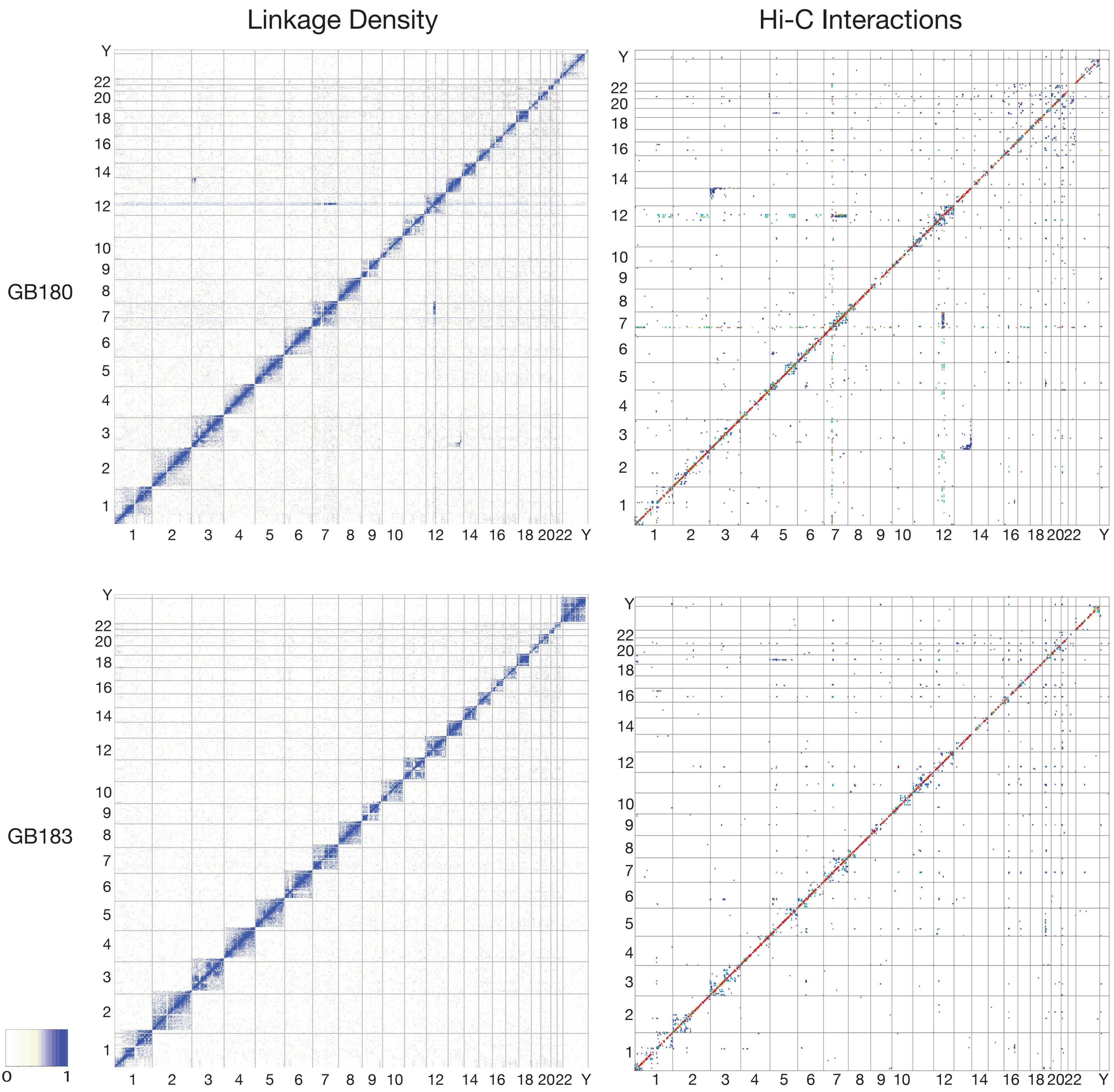

Figure S8: Linkage density plots and Hi-C interaction heatmaps for tumours GB180 and GB183.
Plots of normalised linkage densities (left) and Hi-C interaction heatmaps generated using 500 kb probes (right) for tumours GB180 (top) and GB183 (bottom).

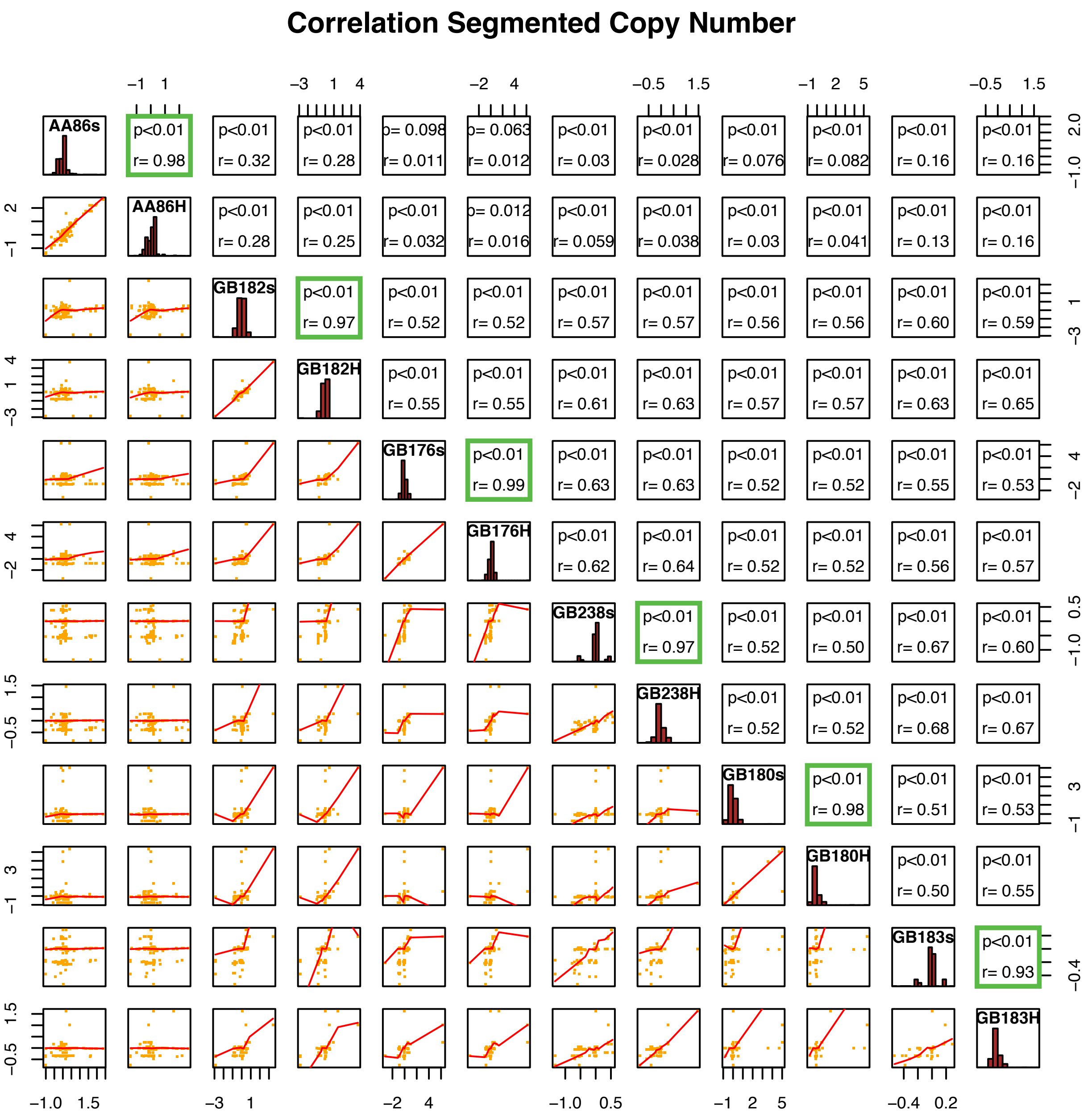

Figure S9: Correlation of QDNAseq segmented copy number results for sWGS and Hi-C. Correlation coefficients for QDNAseq results (segmented copy number) for sWGS and corrected Hi-C inputs for the six brain tumours tested. Boxes outlined in green show r values (p<0.01) for the sWGS and Hi-C results for the tumour named in the adjacent box.

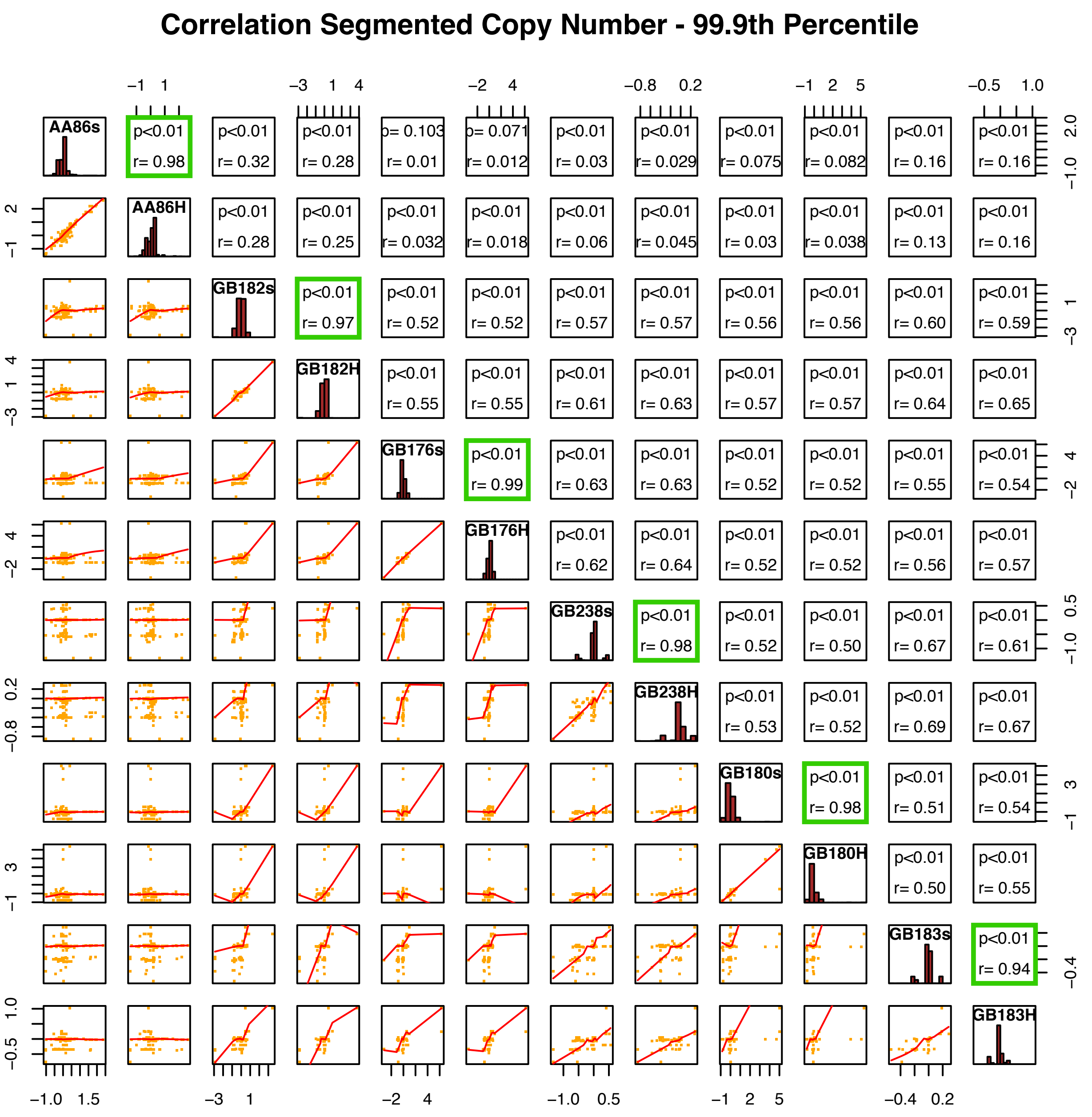

Figure S10: Correlation of QDNAseq segmented copy number results for sWGS and Hi-C using the 99.9th percentile cutoff.

Correlation coefficients for QDNAseq results (segmented copy number) for sWGS and corrected Hi-C inputs with bins having total difference values (sum of difference between sWGS and Hi-C results for all six tumours) falling above the 99.9th percentile being excluded. Boxes outlined in green show r values (p<0.01) for the sWGS and Hi-C results for the tumour named in the adjacent box.

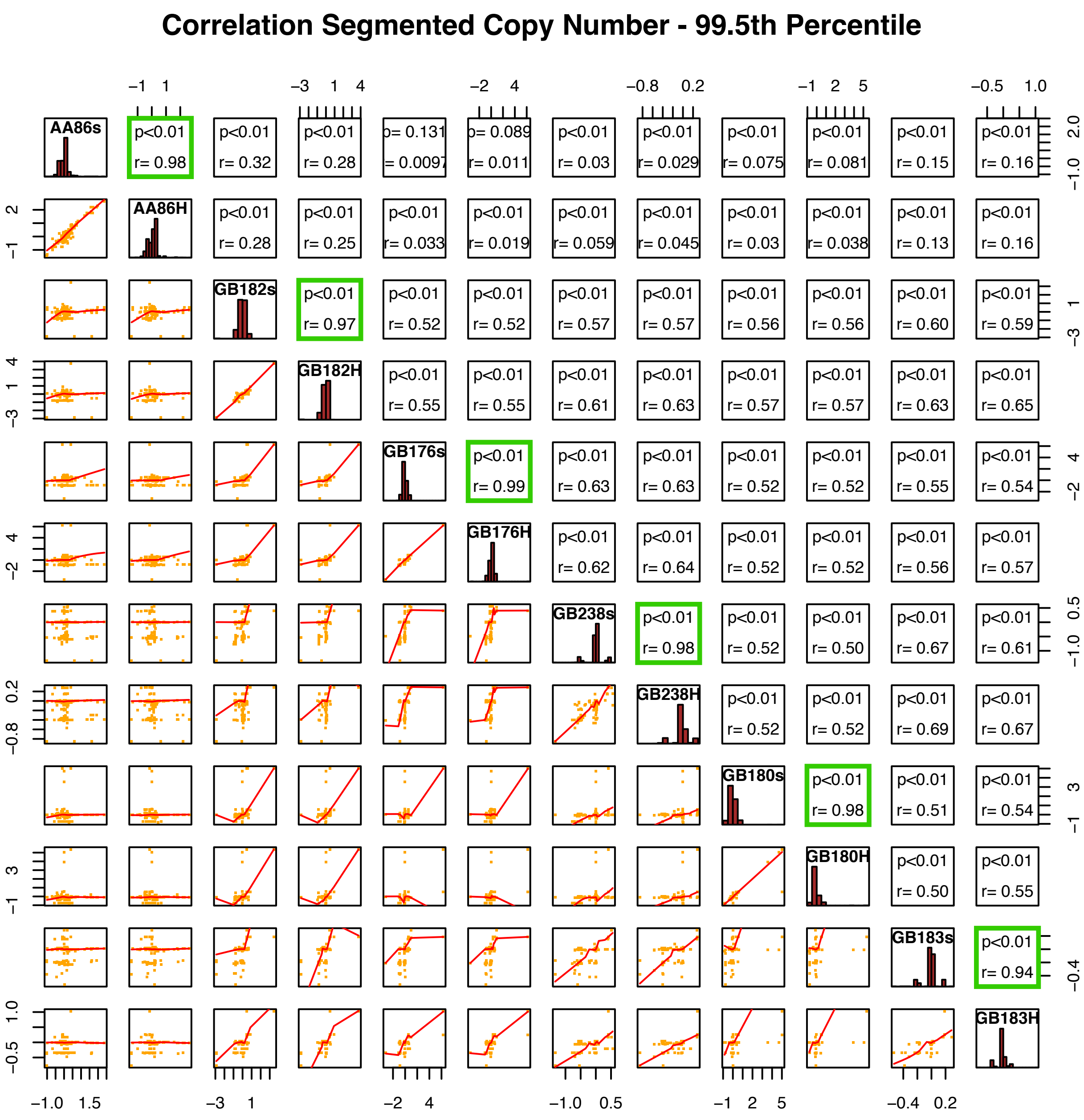

Figure S11: Correlation of QDNAseq segmented copy number results for sWGS and Hi-C using the 99.5th percentile cutoff.

Correlation coefficients for QDNAseq results (segmented copy number) for sWGS and corrected Hi-C inputs with bins having total difference values (sum of difference between sWGS and Hi-C results for all six tumours) falling above the 99.5th percentile being excluded. Boxes outlined in green show r values ($p<0.01$) for the sWGS and Hi-C results for the tumour named in the adjacent box.
