# Supplementary material for: Hi-C as a tool for precise detection and characterisation of chromosomal rearrangements and copy number variation in human tumours
Source: Genome Biol. 2017 Jun 27;18:125. doi: 10.1186/s13059-017-1253-8 (PMC5488307; doi:10.1186/s13059-017-1253-8)
Supplement: Supplementary file 5 — Read counts and sequence coverage of processed samples and Table S5: GEO sample accession numbers. (DOCX 90 kb) [file 13059_2017_1253_MOESM5_ESM.docx]

**Table S4: Read counts and sequence coverage of processed samples**

|  |  |  | No.  Reads | Read Pairs | Valid Read Pairs | Mean Coverage |  |
| --- | --- | --- | --- | --- | --- | --- | --- |
| Hi-C | **Tumours** | GB176 | 38531962 | 25850981 | 17587863 | 0.55 |  |
|  |  | GB182 | 31369053 | 21540944 | 15685839 | 0.49 |  |
|  |  | GB183 | 25472806 | 17342251 | 12388094 | 0.39 |  |
|  |  | GB180 | 29370451 | 19518376 | 11776559 | 0.37 |  |
|  |  | AA86 | 28711290 | 18024005 | 11184282 | 0.35 |  |
|  |  | GB238 | 32061085 | 20563626 | 9410957 | 0.30 |  |
|  |  | | | | | | |
|  |  | | | | | |  |
|  | **Cells** | DD1618 | 41622415 | 28451875 | 12835769 | 0.40 |  |
|  |  | FY1199 | 24201879 | 16763888 | 10958071 | 0.35 |  |
|  |  | GM07017 | 24690448 | 16767297 | 9188769 | 0.29 |  |
|  |  |  |  |  |  |  |  |
| QDNAseq | **sWGS** | GB176 | 15694045 | N/A | N/A | 0.23 |  |
|  |  | GB182 | 59651072 | N/A | N/A | 0.89 |  |
|  |  | GB183 | 33879567 | N/A | N/A | 0.49 |  |
|  |  | GB180 | 44028469 | N/A | N/A | 0.64 |  |
|  |  | AA86 | 49958867 | N/A | N/A | 0.71 |  |
|  |  | GB238 | 46398260 | N/A | N/A | 0.67 |  |
|  |  | | | | | | |
|  |  | | | | | |  |
|  | **Hi-C** | GB176 | 72188264 | N/A | N/A | 1.14 |  |
|  |  | GB182 | 58984775 | N/A | N/A | 0.95 |  |
|  |  | GB183 | 47738132 | N/A | N/A | 0.76 |  |
|  |  | GB180 | 55272023 | N/A | N/A | 0.88 |  |
|  |  | AA86 | 53061786 | N/A | N/A | 0.83 |  |
|  |  | GB238 | 60235745 | N/A | N/A | 0.97 |  |
|  |  |  |  |  |  |  |  |

Read counts and mean sequence coverage for Hi-C (top) and QDNAseq (bottom) experiments for human samples. N/A = Not applicable.

**Table S5: GEO sample accession numbers**

| Sample | Experiment Type | Sequencing Type | Species | GEO Sample No. |
| --- | --- | --- | --- | --- |
| GB176 | HiC | PE50 | Human | GSM2176966 |
| GB182 | HiC | PE50 | Human | \| GSM2176968 \| \| --- \| |
| GB183 | HiC | PE50 | Human | GSM2176969 |
| GB180 | HiC | PE50 | Human | GSM2176967 |
| AA86 | HiC | PE50 | Human | GSM2176962 |
| GB238 | HiC | PE50 | Human | GSM2176970 |
| DD1618 | HiC | PE50 | Human | GSM2176963 |
| FY1199 | HiC | PE50 | Human | GSM2176965 |
| GM07017 | HiC | PE50 | Human | GSM2176971 |
| EKLF-/- | HiC | PE125 | Mouse | GSM2176964 |
| GB176 | sWGS | SE50 | Human | GSM2176966 |
| GB182 | sWGS | SE50 | Human | \| GSM2176968 \|  \| \| --- \| --- \| |
| GB183 | sWGS | SE50 | Human | GSM2176969 |
| GB180 | sWGS | SE50 | Human | GSM2176967 |
| AA86 | sWGS | SE50 | Human | GSM2176962 |
| GB238 | sWGS | SE50 | Human | GSM2176970 |

GEO sample accession numbers for the datasets used. PE=Paired end. SE=Single end. sWGS=shallow whole genome sequencing.
